# Early human embryos are naturally aneuploid—can that be corrected?

**DOI:** 10.1007/s10815-016-0845-7

**Published:** 2016-11-29

**Authors:** Amy Lee, Ann A. Kiessling

**Affiliations:** 1 Division of Reproductive Endocrinology, Department of Obstetrics and Gynecology, Massachusetts General Hospital, Boston, MA USA; 2 Bedford Research Foundation, Bedford, MA USA

**Keywords:** Human embryo, Chromosome, Euploid, Aneuploid, In vitro fertilization, Preimplantation genetic screening, Egg donor

## Abstract

Aneuploidy is common and may be a natural occurrence in early human embryos. Selecting against embryos containing aneuploid cells for embryo transfer has been reported to increase clinical pregnancies per transfer in some studies, but not others. Some aneuploidy is due to misallocation of chromosomes during meiosis, in either the egg or sperm, but most aneuploidy is due to misallocation of chromosomes during mitoses after fertilization. Big questions are as follows: Why does this happen? How much aneuploidy in a preimplantation embryo is compatible with normal fetal development? Is aneuploidy increased by in vitro culture, and/or could it be prevented or corrected in the IVF lab?

## Background

Signaling the mother that it is developing is arguably the single most important activity of a fertilized human egg. The signal must be timely and robust, doubling at least every 2 to 3 days. The best known signal is hCG, but there are probably others, such as IL4 and IL17b [1].

Without robust, timely, embryo-to-mother signals, a miscarriage occurs. This no compromise system conserves maternal resources so precious uterus time is not wasted on a defective conception—which is common in humans. Such conservation of maternal resources is also observed in other placental mammals, such as mice and sheep. Transfer of one embryo into a mouse foster mother almost always results in miscarriage; mice are designed to carry multiple fetuses, so signals from a single fertilized egg are not sufficient to maintain her pregnancy. Similarly with goats and sheep, they almost always have at least twins, suggesting singleton conceptions do not generate enough pregnancy signal to maintain a pregnant state.

How does a fertilized egg accomplish increasing signals to the mother? Starting with only one full set of chromosomes, there are three possibilities: (1) redundant copies of the maternal-signaling genes are quickly synthesized, an hypothesis in keeping with Nobel Laureate Howard Temin’s views [2], and supported by frog development in which multiple copies of ribosomal genes are synthesized early after fertilization [3]; or (2) transcription and translation from the maternal-signaling genes are preferentially enhanced immediately after fertilization; or (3) the entire set of chromosomes is doubled at least daily, thus doubling the gene dose needed to produce the embryo-to-mother signal.

In scenario (1), redundant copies of maternal-signaling genes have not shown up on the many genomic hybridization studies that have now been reported for human embryos [4] so that seems less likely, although not proven wrong.

Evidence in support of scenario (2), specific-enhanced transcription and translation of maternal-signaling genes, is lacking, although this attractive possibility is worthy of further study.

Human eggs undergo the first cell division by approximately 30 h after insemination. The second cell division to four cells occurs approximately 16 h later, thus accomplishing a fourfold increase in gene copy number within 48 h [5]. On the third day, the embryo doubles the copy number again to eight cells which continue to divide and compact into a morula. This rate of chromosome doubling supports scenario (3) that the necessary increase in embryo-to-maternal signal may be accomplished by corresponding increases in gene copy number. To facilitate these rapid divisions, there is over-expression of multiple cell cycle-promoting genes, such as cyclin E, Myc, and the Aurora kinases [6] and the absence of cell cycle check points, RB and WEE1 [7, 8].

On day 5 or 6, the human embryo expands into the blastocyst separating into the inner cell mass and the trophectoderm. At this stage, in vitro developed human embryos are comprised of approximately 60 cells, in keeping with cell doublings at least every 24 h following fertilization. If human embryo development is slowed in vitro, as is the case in mouse embryos [9], in vivo developed human embryos would be 120 cells at the blastocyst stage, with cell doublings every 18 h.

The lack of cell cycle check points renders the early embryo susceptible to possible errors in DNA synthesis and unequal distributions of chromosomes during cleavage divisions. RB, the retinoblastoma gene, is one of the first reported human oncogenes [10] and is responsible for cell cycle arrest in G1, the cell cycle stage in which most somatic cells come to rest. Cells are brought out of G1 arrest by growth factor stimulation of cyclins that, in combination with their respective kinases, overcome the RB blockade and promote progression to DNA synthesis. Cells lacking RB, such as cancer cells, do not require growth factors to stimulate cell division. The limited detection of canonical growth factor receptor messages on microarrays of 8-cell human embryo RNAs [11] further supports the lack of a growth factor requirement for human blastomeres to proceed through gap 1, raising the question of what, if any, cellular controls are in place to ensure the faithful replication of the chromosomes of the early embryo? A newly discovered tumor suppressor gene, UHRF2, detected at elevated levels on the 8-cell embryo microarrays [8] and more recently on human oocyte microarrays [12], is an attractive candidate. UHRF2, a nuclear protein frequently lost in cancer cells [13] and downregulated in fully differentiated cells, appears to be a central player in the cell cycle by coordinating the ubiquitin-protease system with methylated DNA. It has been shown to bring about the degradation of cyclins D1 and E1, effecting a G1 arrest, and interacts specifically with hemi-methylated DNA [14]. The discovery of UHRF2 expression in 8-cell human embryos lacking RB suggests a unique role for this interesting nuclear protein that apparently connects several cell cycle processes with the methylation state of the DNA.

Over-expression of geminin may guard against errors in DNA replication during S phase [6]. The silence of the G2 checkpoint, WEE1, raises concerns about what elements are in place to support accurate allocation of chromosomes to daughter cells. High levels of detection of aurora kinase mRNAs, major players in centrosome duplication and formation of the mitotic spindle, suggest rapid progress through G2. Kinase activities may be modulated by over-expression of Incenp (inner centrosome passenger protein) and Stag proteins, included in cohesion, and the Pttg proteins to block separase activity, to allow sufficient time for accurate chromosome attachment and line up at anaphase. Nonetheless, the over-expression of aurora kinase genes, in combination with over expression of cell cycle drivers such as cyclin E and Myc, is a recipe for unequal chromosome allocation to daughter cells, and cytoplasmic fragments.

The dependence of the human egg on the fertilizing sperm to be the source of the centrosome, responsible for the two centrioles needed to generate the spindle microtubules for the first mitotic division, emphasizes the importance of the sperm for the first euploid cleavage, a couple of cell cycles before the onset of paternal gene expression [15].

## Embryonic aneuploidy

A systematic review and meta-analysis of chromosomal studies of human embryos in 2011 reported that 73% of all human embryos resulting from IVF contain some aneuploid cells [16, 17]. What are the consequences to the early embryo of an unequal distribution of chromosomes among the blastomeres? The answer to this important question is not simple. Approximately 20% of human eggs and 9% of human sperm are thought to be aneuploid [18]. If embryo aneuploidy is due to an error in meiosis, estimated to account for about 25% of observed embryo aneuploidy [19], it will persist and affect every blastomere, unless aneuploidy-correcting mechanisms can be activated. Sperm meiosis occurs continually in the adult male testis, and circumstances contributing to misallocation of chromosomes during male meiosis are not understood. The development of methods of evaluating the chromosome content of individual sperm heads have revealed a measurable incidence of chromosome loss in sperm [20], suggesting failure during meiosis to capture all the chromosomes into new nuclei following anaphase.

Egg meiosis resumes during the final stages of egg maturation, just prior to ovulation. Half of the egg chromosomes are extruded into the first polar body, completing meiosis I, and the egg immediately enters meiosis II, arresting at metaphase II until fertilization. The metaphase arrest is due to the action of the protooncogene kinase, cMos [21]. Sperm entry into the cytoplasm leads to a reduction in cMos activity, allowing chromosome separation and the extrusion of the second polar body. Errors in anaphase at either meiosis I or meiosis II have been shown to occur more frequently with advancing maternal age [22]. Some studies, but not others, have suggested that the ovulation induction protocols used for standard IVF procedures may increase the frequency of meiotic errors in eggs, thus secondarily increasing aneuploidy in the resulting embryos [23, 24].

If the newly fertilized egg is missing a chromosome from either the sperm or the egg, it would need to be specifically duplicated to give rise to euploid blastomeres. If three copies of any chromosome are present, one would need to be eliminated, or the fertilized egg would have to silence one chromosome copy to avoid over-expression of those genes, perhaps in a manner analogous to the X chromosome inactivation that occurs in female embryo cells. Aneuploidies in which only one copy of a chromosome from either sperm or egg is present are apparently not compatible with fetal development, with the exception of the X chromosome. Turner’s syndrome, in which females have a single X chromosome, occurs in 1 in 2000 female births. The majority of Turner’s patients have the maternal X chromosome [25, 26], suggesting the fertilizing sperm was missing a sex chromosome. In contrast, aneuploidies with three copies of one chromosome support some fetal development, such as trisomy 13, 18, and 21, although only trisomy 21 (Down’s syndrome) is actually compatible with development into adulthood.

**Fig. 1:**
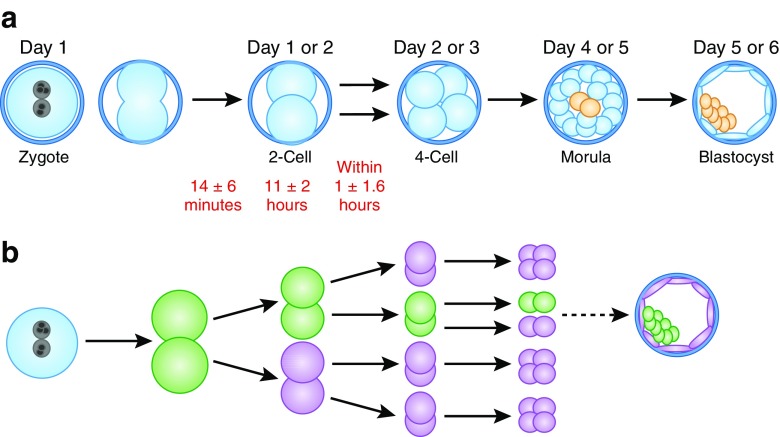
Early human development. **a** The zygote possesses one pronucleus containing egg chromosomes and another pronucleus containing sperm chromosomes. Both sets of chromosomes are duplicated before the first cleavage to two cells. The morula forms at the 8- to 16-cell stages, trapping one or two cells inside that undergo commitment to become the inner cell mass (ICM) within the blastocyst. The ICM gives rise to the fetus. The outer cells of the blastocyst become committed to trophoblast, precursor to the placenta. **b** Theoretical aneuploidy in early development. The scheme depicts the highest rate of aneuploidy (*purple cells*) that could form the ICM from a euploid cell (*green*) and produce a normal fetus. This theory is supported by several lines of evidence, including chromosomal analyses that reveal both aneuploid and euploid cells in human blastocysts

Aneuploidy arising during the mitoses that follow fertilization are thought to account for the majority of the aneuploidy observed in preimplantation human embryos. The most likely anaphase errors appear to be loss of a chromosome poorly attached to the spindle that does not become enclosed within the daughter nucleus, and failure of a chromosome to separate at anaphase. The loss of a chromosome results in one euploid and one aneuploid cell, whereas failure to separate at anaphase leads to two aneuploid cells, one with too many chromosomes and one with too few [15, 17]. It can be argued that the blastomeres contributing to the inner cell mass that will give rise to the fetus need to be euploid to support normal fetal development (Fig. 1). If true, it may be important that the first cleavage to two cells is euploid to generate a population of euploid cells. But, should chromosome-correcting pathways be operational, even this may not be necessary.

Commitment to inner cell mass versus trophoblast begins at approximately the 16-cell stage when one or two cells become positioned within the ball of blastomeres (Fig. 1). Hence, during most preimplantation stages, on the order of only 10% of total cells will give rise to the fetus. Given the polyploidy and endoreduplication among trophoblast cells [27], aneuploidy among that cell population may not negatively influence further development.

## Embryonic genome analyses

Several lines of evidence support the notion that aneuploidy is common, maybe universal, in early stage mammalian embryos. One is the very small fraction of individual blastomeres from mouse and human embryos competent to continue dividing into a stable line of stem cells. In 2006, Lanza and colleagues biopsied human embryos and reported 2 of 91 blastomeres developed into embryonic stem cell lines [28]. Two years later, van de Velde and colleagues dissociated four human embryos at the 4-cell stage (16 blastomeres) only two of which (12%) developed into embryonic stem cell lines, one normal XY and one mosaic XX [29]. In 2007, Wakayama and colleagues attempted to isolate mouse embryonic stem cell lines from individual mouse blastomeres. From six 2-cell stage embryos, both blastomeres developed into embryonic stem cell lines from one embryo; one blastomere, but not the other, from two embryos; and none from the remaining three embryos. If aneuploidy accounts for the failures, 16% of the 2-cell mouse embryos were euploid. From three 4-cell stage embryos, only one blastomere developed into an embryonic stem cell line (8%), and from six 8-cell embryos, four blastomeres from four different embryos (8%) developed into embryonic stem cell lines. In contrast, the same group developed 14 new mouse embryonic stem cell lines from the inner cell masses of 16 blastocysts.

One explanation for the inability of single blastomeres to give rise to stable stem cells lines is that cells of the early embryo require contact with each other for continued development, but an equally plausible explanation is that fewer than 20% of the individual blastomeres from mouse and human eggs are truly euploid and capable of sustaining independent continued cell division.

Another line of evidence that aneuploidy is common stems from chromosome analyses of individual blastomeres biopsied from human embryos at various stages of early development. The original goal of the early embryo biopsies was to analyze the embryo for sex chromosomes, to avoid sex-linked genetic defects in offspring or specific gene mutations associated with disease, such as Tay Sachs, cystic fibrosis, or sickle cell anemia [30–32]. Several research groups reported removal of one or two blastomeres from early cleaving human embryos for genetic analyses without apparently reducing the viability of the embryo itself [33]. Initially, DNA amplification with polymerase chain reaction was utilized to detect Y chromosomes or obtain gene sequences, and soon thereafter, chromosome analyses were performed on individual blastomeres by fluorescence in situ hybridization (FISH).

Because chromosomal analyses of miscarriages demonstrated that aneuploidy is associated with early pregnancy failures [34, 35], the blastomere biopsy FISH techniques were quickly applied to assessing aneuploidy in individual cells of early embryos in order to transfer only euploid embryos. Gianaroli and colleagues showed improvements in implantation frequencies per embryo transfer by deselecting the aneuploid embryos in poor prognosis patients [36]. Although initially promising, single cell biopsy of early cleaving embryos and analysis by FISH of six to eight chromosomes limited this technology, but supported the notion that aneuploidy is common in preimplantation embryos. Knowing this, however, did not seem to improve overall pregnancy outcomes. In a multi-center, randomized, controlled trial, Matstenbrooke and colleagues found that PGS with FISH lowered ongoing pregnancies by 12%. In 2007, the American Society of Reproductive Medicine stated “available evidence does not support the use of PGS as currently performed to improve live birth rates in patients with advanced maternal age, previous implantation failure, or recurrent pregnancy loss” [37, 38].

Many of the limitations in early aneuploidy screening by FISH have been overcome. Culturing the embryo longer permits trophectoderm (TE) biopsy, which allows sampling of more cells and is better tolerated by the embryo. This certainly improves the accuracy of detecting defects in specific genes, but may not reflect the chromosome status of the developing fetus. Molecular biology techniques, such as real-time PCR, single nucleotide polymorphism (SNP) analyses, comparative genomic hybridization (CGH), or, more recently, next generation sequencing (NGS), have been applied to genetic analyses of three to five trophoblast cells and shown to be more reliable in detecting chromosomes than FISH [4, 39]. Real-time PCR (qPCR) quantifies chromosome copy number of each chromosome to a reference tissue known to be euploid. Both SNP and CGH microarrays compare amplified blastomere DNA to a reference tissue known to be euploid; the readout shows deletions and duplications as a higher or lower signal compared to the control. More recently, in NGS, the fragmented DNA is amplified in clusters and sequenced. The sequences are then aligned with bioinformatics software to reveal deletions or amplifications of specific chromosomes [40].

The development of more global methods to assess chromosome number has led to more widespread screening of embryos for aneuploidy. A 2010 comparison of FISH with SNP analyses of 13 embryos by Richard Scott and colleagues revealed that 8 of the 13 appeared euploid by SNP analyses and none were euploid by FISH analyses, confirming not only the high (at least 38%) incidence of aneuploidy [39], but the superior accuracy of SNP analyses over FISH analyses. In a follow-up study, 50 embryos judged to be aneuploid by FISH at early cleavage stages were allowed to develop further and re-analyzed at the blastocyst stage by SNP. Only 21 (42%) were aneuploid by SNP analyses with no apparent preference for inner cell mass or trophoblast cells [41], supporting the notion that SNP analyses provided a more accurate assessment of chromosome content, but also suggesting some aneuploidy-correcting mechanisms may exist in early cleavage stage embryos.

The ability to genetically screen a few cells from human embryos without apparently reducing their developmental potential immediately led to the question of whether transfer of only embryos testing euploid would improve pregnancy rates. This question has spawned numerous studies.

In an early study, aneuploidy diagnosed by CGH on biopsy of both blastomere and trophectoderm biopsies resulted in 96% of transferred embryos failing to implant [42]. Schoolcraft et al., found 51% of blastocysts of high-risk individuals (advanced age, history of IVF failures, or spontaneous abortion) were aneuploid. And when euploid embryos were transferred, the pregnancy rate was 82% despite transferring fewer embryos in subsequent cryothaw cycles [43]. In a randomized controlled trial, 155 patients with at least two blastocysts were randomized on day 5. The clinical implantation rate was 80% in the CGH group compared to 63% in untested controls (RR = 1.26, *P* = 0.002) and the live birth rate was 66% in the CGH group compared to 48% in untested controls (RR = 1.39, *P* = 0.001) [19]. Further studies have shown similar results in good prognosis patients. The Blastocyst Elective Selective Transfer (BEST) trial evaluated the outcomes of 175 good prognosis patients randomized to aneuploidy screening with elective single embryo transfer (eSET) or double embryo transfer chosen by morphology alone. The incidence of ongoing pregnancies per embryo transfer was similar between the two groups (61 vs. 65%, (RR = 0.9, CI 0.7–1.2) [44]. The cumulative delivery rates after fresh and first frozen cycles were similar in both groups, 65 vs. 72%(*P* = 0.6) with a drastic reduction in multiple births 1.6% compared to 47% (*P* < 0.001) with reductions in preterm delivery, low birth rate, and NICU admission [45].

## The future

Acceptance of the fact that aneuploidy is common, perhaps normal, in early cleaving human embryos will lead to the development of paradigms about how much and what types of aneuploidy are incompatible with further development. The normal development of identical twins illustrates that only 50% of cells in an early embryo are needed for normal development to offspring.

Data that support the existence of cellular mechanisms in blastomeres that correct aneuploidy are limited, although a recent report of haploid human parthenogenetic stem cells converting to diploid stem cells during culture demonstrate that robust chromosome copy number correcting mechanisms develop at some point [46]. In fact, it is difficult to maintain a line of haploid cells. Not surprising, they are smaller and divide more slowly and never seem to reach 100% of the cell population. To maintain a haploid state for studies, the researchers needed to re-sort the cell population to select for haploid genotypes every few cell doublings [43].

Similarly, data that support aneuploidy-correcting mechanisms are also lacking—do those cells simply undergo programmed cell death at some point during development as is the case for frog embryos [47]? Does chromothripsis occur in early human embryos? Revealed by analyses of whole genome sequences of several cancer genome datasets, chromothripsis is a complex process that includes chromosome duplications, deletions, translocations, and inversions [15].

It may never be possible to fully assess the incidence of aneuploidy in human embryos developing in utero, but the degree of aneuploidy reported in spontaneous miscarriages indicates it certainly exists, and increases with advancing maternal age. The possibility of designing conditions that suppress aneuploidy during early cleavage stages in vitro is an intriguing one. The inclusion of small molecule inhibitors of the aurora kinases in culture medium to at least cause a pause during gap 2 is one possibility [48]. It is also possible that an as yet to be discovered circadian signal helps control the cell cycle, a possibility revealed by the detection of core circadian oscillators on the microarrays of 8-cell human embryos [8]. More recent pilot studies of early cleavage stage mouse embryos have revealed circadian expression of period 2 (manuscript in preparation), supporting a possible novel role for this core oscillator in early development.

Another approach is the inclusion of messenger RNA constructs that encode naturally occurring, but perhaps limited, cell cycle check points, such as UHRF2, described above, that may stabilize the onset of S phase. BUB1, a kinase active in G2 that lines up on the kinetochore, opposes aurora kinases to delay the onset of anaphase and help ensure proper chromosome segregation [49]. Suitable mRNA constructs microinjected into eggs at the time of ICSI would be translated during the ensuing couple of cell cycles before being degraded. BUB1B was robustly detected on the microarrays of fresh, normal morphology 8-cell human embryos [8] but could be lacking in fragmented, more aneuploid embryos.

Two decades of research on the incidence of aneuploidy in human embryos has clearly documented that the majority, if not all, embryos have some aneuploid blastomeres at some time in early development, with the incidence increasing with advanced maternal age [15–17]. There may be no benefit to screening embryos from young donor eggs. The next decade needs to focus on what aneuploid state is compatible with normal fetal development, and what, if anything, can in vitro fertilization laboratories do to adjust early development toward the necessary euploid complement of cells for robust embryonic development to healthy offspring. The majority of the cell cycle, DNA synthesis, and growth factor family genes over-expressed in 8-cell human embryos have more recently been reported to be circadianly expressed in some mouse tissues [50]. This is in keeping with the noted over-expression of the core circadian oscillators in 8-cell embryos relative to cultured pluripotent stem cells [8]. Although primary cells in culture appear to maintain their circadian oscillations for up to 2 weeks [51], newly fertilized eggs may benefit from some, as yet unknown, circadian signal support for the week they are commonly now held in culture.

It is possible that IVF laboratories may be able to develop corrective measures to alleviate the natural tendency toward aneuploidy exhibited by human conceptions in their attempt to rapidly multiply their genetic information.
